# Impact of education in patients undergoing physiotherapy for lower back pain: a level I systematic review and meta-analysis

**DOI:** 10.1007/s00068-025-02788-9

**Published:** 2025-02-19

**Authors:** Filippo Migliorini, Nicola Maffulli, Luise Schäfer, Nicola Manocchio, Michela Bossa, Calogero Foti, Marcel Betsch, Joshua Kubach

**Affiliations:** 1 Department of Life Sciences, Health, and Health Professions, Link Campus University, Via del Casale Di San Pio V, 00165 Rome, Italy; 2 Department of Orthopedics and Trauma Surgery, Academic Hospital of Bolzano (SABES-ASDAA), 39100 Bolzano, Italy; 3 Faculty of Medicine and Psychology, University “La Sapienza” of Rome, Rome, Italy; 4 School of Pharmacy and Bioengineering, Keele University, Faculty of Medicine, Stoke On Trent, ST4 7QB UK; 5 Centre for Sports and Exercise Medicine, Barts and the London School of Medicine and Dentistry, Mile End Hospital, Queen Mary University of London, London, E1 4DG UK; 6 Department of Orthopaedic and Trauma Surgery, Eifelklinik St. Brigida, Kammerbruschstr. 8, 52152 Simmerath, Germany; 7 Clinical Sciences and Translational Medicine Department, Tor Vergata University, 00133 Rome, Italy; 8 Department of Trauma and Orthopedic Surgery, Friedrich-Alexander University Erlangen-Nurnberg, University Hospital Erlangen, Erlangen, Germany

**Keywords:** Lower back pain, Spine, Physiotherapy, Education

## Abstract

**Introduction:**

Lower back pain (LBP) is one of the most common musculoskeletal disorders in modern society, with a lifetime incidence of up to 90%. According to most national and international guidelines, educational interventions play a central role in the multimodal treatment of LBP. This systematic review and meta-analysis investigated the impact of educational interventions on pain and disability in patients with LBP undergoing physiotherapy compared to patients without educational interventions undergoing physiotherapy.

**Methods:**

In October 2024, a comprehensive computer-aided search was performed to assess the online databases PubMed, Web of Science, Google Scholar, and Embase. The search followed the Preferred Reporting Items for Systematic Reviews and Meta-Analyses (PRISMA) criteria with an established PICOTD algorithm. Two authors independently performed the data extraction and risk of bias evaluation. The primary outcome measures extracted were a pain score (VAS or NRS) and the Roland Morris Disability Questionnaire (RMQ).

**Results:**

Data from 8152 patients were retrieved. The mean length of follow-up was 6.2 ± 3.9 months, the mean length of symptom duration was 66.7 ± 51.6 months, and the mean age of the patients was 46.7 ± 9.2 years. Compared to physiotherapy alone, additional education did not reduce pain (P = 0.4) or disability according to the RMQ (P = 0.9).

**Conclusion:**

The addition of education did not impact pain and disability in patients undergoing physiotherapy for chronic non-specific LPB.

**Level of evidence:**

Level I, systematic review and meta-analysis of RCTs.

**Supplementary Information:**

The online version contains supplementary material available at 10.1007/s00068-025-02788-9.

## Introduction

Lower back pain (LBP) is a common musculoskeletal condition characterised by discomfort or pain in the lower region of the spine [1, 2]. When the pathoanatomical cause (e.g. fracture, tumour, inflammation, structural deformity) of the pain cannot be determined, LBP is usually defined as “nonspecific”[3, 4]. LBP can become chronic back pain if it persists over three months or more [5, 6]. It has become a frequent health problem in our society. However, its pathogenesis remains unclear, with multiple factors imposing an increased risk for LBP, such as level of education, psychosocial factors, body mass, smoking status, active lifestyle, mechanical and genetic factors and many more [7, 8]. With reported lifetime incidences of up to 90% [9], LBP is the leading cause of absence from work and one of the most common indications for the prescription of physiotherapy and rehabilitation programs [3, 10–12]. The prognosis for LBP remains challenging. Only 40% of patients recover completely by 12 months of the first episode, with the remaining 60% experiencing varying levels of disability, pain intensity, and recurrent sick leave [5, 13].

Rehabilitation interventions can broadly be classified into educational and non-educational programs [14]. Educational programs involve teaching patients and/or caregivers about their condition, the importance of self-management, and demonstrating strategies to prevent future recurrences [15–17]. On the other hand, non-educational programs involve physiotherapy interventions, such as exercise therapy, manual therapy, and physical agents (i.e. ultrasound therapy) or pharmacological interventions [18–23]. Education is also part of physiotherapy interventions, even without educational modalities. The physiotherapist speaks, instructs and gives advice to the patient. However, the primary focus of different modalities is their own (e.g. motor impairment for a motor re-educational program, improving self-condition awareness for education modalities such as back school). Most national and international guidelines recommend a multimodal approach to counteract the different aspects of chronic LBP, including psychosocial and organic factors, using non-pharmacological and pharmacological treatment options; this multimodal approach is what in the rehabilitation field is called an Individual Rehabilitation Project [24–26].

Several studies have investigated the effectiveness of educational and non-educational programs for treating chronic non-specific LBP [27–30]. In fact, in the current literature, there is a well-recognized roles for patient education and physiotherapy in managing LBP [28, 31]. A comprehensive Individual Rehabilitation Project for LBP should incorporate educational and non-educational components. A classic approach to patient education is the “Back School” program, which includes information on the physiology and pathology of the spine (i.e., postural alterations, articular stiffness, or muscle weakness) and its biomechanics [32, 33]. It also educates patients on the best ergonomy of the spine during daily activities, sports and recreation [32, 33]. Furthermore, it teaches patients to maintain a correct posture while working at a desk or carrying heavy objects [32, 33]. Information is also provided about why specific postures decrease back muscle tension and spinal load during work and daily activities (e.g., dressing, eating, and bathing) [32, 33]. Other important re-educational programs can be represented by various physiotherapy modalities (e.g. exercise therapy, manual therapy, and physical agents). The goal is to empower patients to consistently and independently perform prescribed exercises to reinforce the therapeutic benefits [32–34].

Chronic LBP can not only be related to spinal pathogenesis, but psychological factors, such as fear-avoidance beliefs, wrong coping strategies, and mood alterations, influence it [35, 36]. For this reason, educational programmes are often accompanied by a cognitive-behavioural intervention, which includes extensive counselling and information to emphasise the role of stress and coping strategies, the perception of different stressors or threatening events, one’s ability to control stressors or change the situation, and the management of emotional reactions. Furthermore, psychological interventions such as long-lasting multidisciplinary programs based on cognitive-behavioural therapy (CBT) in patients with chronic LBP on disability, kinesiophobia (i.e. fear-based movement avoidance), pain, and the quality of life (QoL), seem to be superior to an exercise program of similar duration [37–39].

Different lower and higher-quality studies have investigated the role of educational interventions in managing acute and subacute LBP [40, 41]. Patient education is more effective than no intervention in the treatment of LBP. However, some studies show no differences between educational and non-educational programs [5, 27, 30]. This is also contributed by the fact that educational intervention can vary from informational material to structured or unstructured educational sessions to psychotherapy [42]. The role of physiotherapy has also been researched extensively, and an overview of meta-analysis and systematic review has concluded that there are helpful effects in the different forms of physiotherapy. However, the overall evidence was moderate to low [4, 25, 29].

In conclusion, there is no definitive answer as to whether combining an educational program and physiotherapy as part of a multimodal treatment plan for patients suffering from LBP results in superior outcomes in pain, disability and return to work. The presented systematic review and meta-analysis investigated the efficacy of education in patients undergoing physiotherapy for chronic LBP. The outcomes of interest were to assess whether additional patient education was associated with improving pain and disability.

## Methods

### Eligibility Criteria

All the randomised controlled trials (RCTs) that evaluated a physiotherapy program’s efficacy in patients with LBP were accessed. According to the authors’ language capabilities, English, German, Italian, French and Spanish articles were eligible. According to the Oxford Centre of Evidence-Based Medicine [43], only RCTs with level I of evidence were considered. Reviews, opinions, letters, and editorials were not considered. Animals, in vitro, biomechanics, computational, and cadaveric studies were not eligible. Studies reporting non-specific [3] or mechanical [44] chronic LBP were included. The pain was defined as chronic when symptoms persisted for a minimum of three months [20]. For the “physiotherapy” category, all the interventions primarily focused on motor functional restoration, pain reduction, and muscle stiffness improvements (e.g. physiotherapy, physical agents application, manual therapy, stretching, and lumbar stabilisation) were considered. On the other hand, modalities primarily focused on patients’ education (e.g. Back School), which tries to give patients basic knowledge about LBP (e.g. epidemiology, risk factors, development, disorders, therapy), were considered for the “education” category. Only studies with a follow-up from one to 12 months were eligible. Missing quantitative data under the outcomes of interests warranted the exclusion of the study.

### Search strategy

This study was conducted according to the Preferred Reporting Items for Systematic Reviews and Meta-Analyses: the 2020 PRISMA statement [45]. The PICOTD algorithm was established:

- P (Problem): chronic LBP;
- I (Intervention): physiotherapy;
- C (Comparison): education versus non-education;
- O (Outcomes): pain and disability;
- T (Study Type): RCT;
- D (Duration): one to 12 months of follow-up.

In October 2024, the following databases were accessed: PubMed, Web of Science, Google Scholar, and Embase, with no time constraints. The search was restricted to RCTs. The matrix of keywords used in each database is shown in the appendix. No additional filters were used in the database search.

### Selection and data collection

Two authors (L.S. and F.M.) performed the database search. All the resulting titles were screened by hand, and the abstract was accessed if suitable. The full text of the abstracts that matched the topic was accessed. If the full text was not accessible or available, the article was not considered for inclusion. A cross-reference of the full-text bibliography was also conducted to identify additional studies. Disagreements were settled by a third senior author (N.M.).

### Data items

Two authors (L.S. and F.M.) performed data extraction. The following data at baseline were extracted: author and year of publication, journal of publication, number of patients included with related mean age and BMI (kg/m^2^), mean length of symptoms duration before the physiotherapy and the length of the follow-up. Data concerning the following PROMs were collected at baseline and the last follow-up: pain scores and the Roland Morris Disability Questionnaire (RMQ) [46]. For the pain scores, the visual analogue scale (VAS) or numeric rating system (NRS) was used interchangeably [47]. Data were extracted in Microsoft Office Excel version 16.72 (Microsoft Corporation, Redmond, USA). Data categorisation (education versus non-education) was conducted by three academic physiatrists with long experience in LBP (C.F., N.M., and M.B.).

### Assessment of the risk of bias

The risk of bias was evaluated following the guidelines in the Cochrane Handbook for Systematic Reviews of Interventions [48]. Two reviewers (L.S. and F.M.) assessed the risk of bias in the extracted studies. Disagreements were solved by a third senior author (**). RCTs were evaluated using the risk of bias of the software Review Manager 5.3 (The Nordic Cochrane Collaboration, Copenhagen). The following endpoints were evaluated: selection, detection, performance, attrition, reporting, and other biases.

### Synthesis methods

The main author (F.M.) performed the statistical analyses following the recommendations of the Cochrane Handbook for Systematic Reviews of Interventions [49]. The IBM SPSS software version 25 (International Business Machines Corporation, Armonk, USA) was used for descriptive statistics. The arithmetic mean and standard deviation were used. The Review Manager software version 5.3 (The Nordic Cochrane Collaboration, Copenhagen) was used for the meta-analyses. The paired t-test was performed with values of P < 0.05 considered statistically significant. The inverse variance method with mean difference (MD) effect measure was used for continuous data. The Mantel–Haenszel method with odd ratio (OR) effect measure was used for binary data. The CI was set at 95% in all the comparisons. Heterogeneity was evaluated through Higgins-I^2^ and χ^2^ tests. If P_χ2_ > 0.05, no statistically significant heterogeneity was found. If P_χ2_ < 0.05, the heterogeneity was assessed following the values of the Higgins-I^2^. If the Higgins-I^2^ test > 70%, high heterogeneity was found. A fixed effect model was set as default. If high heterogeneity was detected, a random model effect was used. Overall values of P < 0.05 were considered statistically significant.

## Results

### Study Selection

**Fig. 1:**
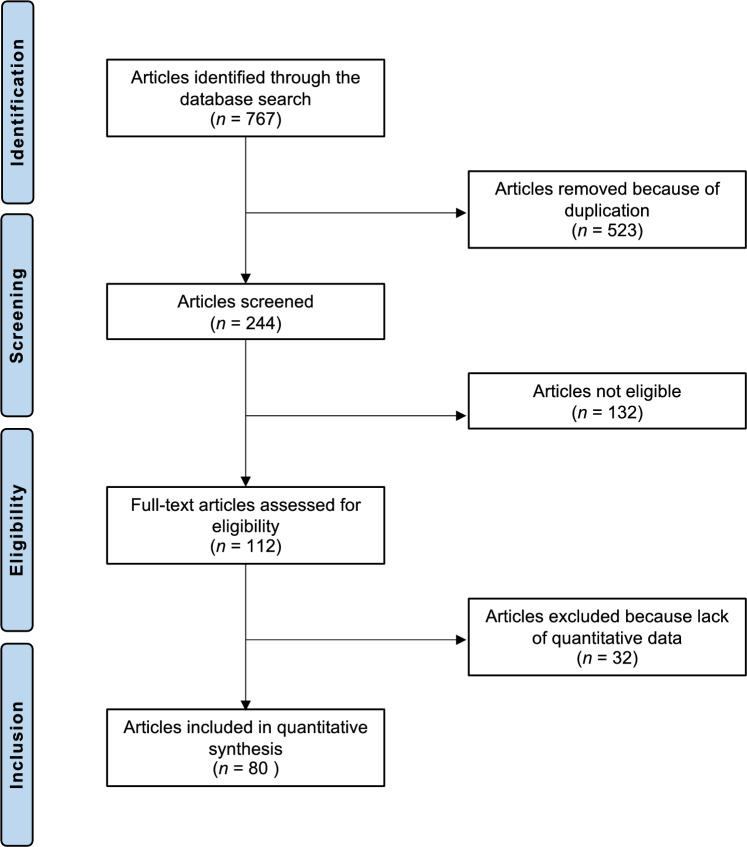
PRISMA flow chart of the literature search

The systematic literature search resulted in 767 articles. A total of 523 were identified as duplicates and, therefore, excluded. A further 132 studies did not meet the defined inclusion criteria and were excluded. In particular, the reasons for ineligibility included inadequate study design (*N* = 62), level of evidence II to V (*N* = 35), not mentioning the therapeutic approaches (N = 13), language limitations (*N* = 17), and follow-up time shorter than one months (*N* = 5). After full-text evaluation, an additional 32 investigations were excluded because they did not offer quantitative data on the outcomes of interest. In conclusion, 80 studies were available for inclusion; all were randomised controlled trials. The results of the literature search are shown in Fig. 1.

### Risk of bias assessment

**Fig. 2:**
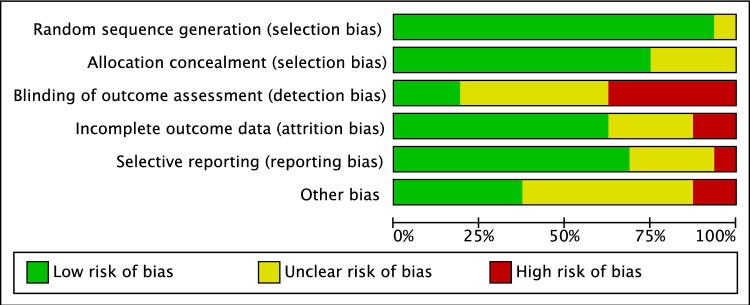
Cochrane risk of bias tool

The risk of bias analysis showed a low risk of selection bias because all included studies were RCTs. The allocation of patients to each treatment group was performed with a high degree of quality in most studies, resulting in a low to moderate risk of allocation bias. Moderate risk was raised for detection and performance bias, attributed to the lack of information on the blinding of investigators and patients during treatment and follow-up. Twenty-two studies did not report information on study dropouts during study enrolment, and the analysis was incompletely reported, resulting in moderate attrition bias. The risk of reporting bias was overwhelmingly low to moderate, and the risk of other biases was mainly low. In summary, the risk of bias graph indicates a good quality methodological assessment of RCTs (Fig. 2) (see Table 1).

**Table 1:** Generalities and patient baseline of the included studies

Author, year	Journal	Treatment	Type Of Movement	Education about LBP	Patients (n)	Mean FU (months)	Mean age	Women (%)
Aasa et al. 2015 [88]	J Orthop Sports Phys Ther	Exercise	Low-Load	Y	25	12	42	54
		Exercise	High-Load Lifting	Y	28		42	57
Added et al. 2016 [89]	J Orthop Sports Phys Ther	Physiotherapy	Individualized	N	74	6	44.6	71.6
		Physiotherapy	Individualized	N	74	6	45.6	71.6
Alfuth et al. 2016 [90]	Orthopäde	Mobilization	Mobilization	N	14	1	50	78.6
		Stabilization	Stabilization	N	13		43	53.9
Areeudomwong et al. 2017 [50]	Musculoskelet Care	Exercise	Contraction	N	21	3	35.4	71.4
		Control		Y	21		36.2	76.2
Azevedo et al. 2018 [51]	Phys Ther	Exercise	Strengthening, Stretching	N	74	4	40.4	58.1
		Control	Various	Y	74		43.4	64.9
Bae et al. 2018 [91]	J Back Musculoskelet Rehabil	Exercise	Core Stabilization	N	18	3	32.7	50
		Exercise	Strenghtening	N	18		32.4	61.1
Balthazard et al. 2012 [92]	BMC Musculoskelet Disord	Spinal Manipulation	High Velocity, Low Amplitude	Y	19	6	44	36
		Physical Agents	Ultrasound	Y	18		42	30
Branchini et al. 2015 [81]	F1000research	Spinal Manipulation	Pressure	N	11	3	48	63.6
		Control	Individualized	N	13		44	69.2
Bronfort et al. 2011 [52]	Spine J	Exercise	Various	Y	101	9	45.6	58.4
		Spinal Manipulation	High-Velocity, Low-Amplitude	N	100		45.2	66
		Exercise	Strengthening	N	100		44.5	57
Cai et al. 2017 [93]	Med Sci Sports Exerc	Exercise	Resistance	N	25	4	28.9	50
		Exercise	Contraction	N	24		26.1	
		Exercise	Stabilization	N	25		26.9	
Cecchi et al. 2010 [94]	Clin Rehabil	Back School	Individualized	Y	68	12	57.9	70
		Physiotherapy	Individualized	Y	68		60.5	61.4
		Spinal Manipulation	Mobilization, Manipulation	Y	69		58.1	68.6
Costa et al. 2009 [95]	Phys Ther	Motor Control Exercise	Individualized	N	77	10	54.6	58
		Sham		N	77		52.8	62
Cruz-Díaz et al. 2015 [96]	Disabil Rehabil	Pilates	Individualized	N	53	10.5	69.6	100
		Physiotherapy	Various	N	48		72.7	100
Dadarkhah et al. 2021 [97]	J Natl Med Assoc	Exercise	Core Stabilization	N	28	12	49	57.1
		Exercise	Core Stabilization	N	28		50	57.1
De Oliveira et al. 2020 [98]	J Physiother	Spinal Manipulation	High-Velocity	N	71	5	45	77
		Spinal Manipulation	Various	N	72		45	78
Diab et al. 2013 [99]	J Back Musculoskelet Rehabil	Exercise	Traction	N	40	6	46.3	45
		Exercise	Stretching	N	40		45.9	42.5
Friedrich et al. 1998 [100]	Arch Phys Med Rehabil	Exercise	Various	N	44	12	43.3	56.8
		Exercise	Various	N	49		44.9	44.9
Frost et al. 1995 [101]	BMJ	Exercise	Aerobics	Y	36	6	34.2	52.8
		Control		Y	35		38.5	51.4
Fukuda et al. 2021 [102]	Braz J Phys Ther	Spinal Manipulation	Joint Mobilzation	N	35	12	35.2	52.9
		Spinal Manipulation	Joint Mobilization, Strengthening	N	35		40.2	
Garcia et al. 2013 [103]	Phys Ther	Back School		Y	74	6	54.2	68.9
		Mckenzie	Symptom Guided	Y	74		53.7	78.4
Garcia et al. 2018 [104]	BMJ	Mckenzie	Various	Y	74	10.75	57.5	78.4
		Control		Y	73		55.5	74
Gardner et al. 2019 [105]	BMJ	Exercise	Individualized	Y	37	10	44	65.8
		Control	Various	Y	38		45	48.6
Gavish et al. 2015 [106]	Physiotherapy	Exercise	Oscillation	N	18	0.75	53.2	33
		Control		N	18		47.1	56
Ghroubi et al. 2007 [107]	Ann Readapt Med Phys	Spinal Manipulation	Symptom Guided	N	32	1	39.1	84
		Sham		N	32		37.4	75
Grande-Alonso et al. 2019 [108]	Pain Med	Multidisciplinary	Various	Y	25	3	39.9	56
		Multidisciplinary	Stabilization	Y	25		38.3	56
Haas et al. 2014 [82]	Spine J	Sham	Various	N	95	10.6	40.9	49
		Spinal Manipulation	High Velocity, Low Amplitude	N	99		41.4	49
		Spinal Manipulation	High Velocity, Low Amplitude	N	97		41.8	49
		Spinal Manipulation	High Velocity, Low Amplitude	N	100		41.2	52
Halliday et al. 2019 [54]	Physiotherapy	Mckenzie	Symptom Guided	Y	35	10	48.8	80
		Motor Control Exercise	Contraction	N	35		48.3	80
Halliday et al. 2016 [53]	J Orthop Sports Phys Ther	Mckenzie	Symptom Guided	Y	32	2	48.8	80
		Motor Control Exercise	Contraction	N	30		48.3	80
Harts et al. 2008 [109]	Aust J Physiother	Exercise	High Intensity	N	23	4	44	0
		Exercise	Low Intensity	N	21		42	
		Control		N	21		41	
Helmhout et al. 2004 [110]	Spine	Exercise	High-Intensity Strengthening	N	41	9	41	0
		Exercise	Low-Intensity Strengthening	N	40		40	
Hicks et al. 2016 [111]	Clin J Pain	Control	Various	N	31	3	69.5	51.6
		Exercise	Stabilization	N	26		70.7	58.1
Hohmann et al. 2018 [112]	Dtsch Arztebl Int	Hirudotherapy		Y	25	2	59.3	88
		Exercise	Various	Y	19	1	56.5	95
Huber et al. 2019 [113]	BMC Musculoskelet Disord	Walking	Walking	N	27	13.5	52.9	51.9
		Walking	Walking, Heat	N	26		53.4	53.8
		Control		N	27		43.8	63.0
Jarzem et al. 2005 [114]	J Musculoskelet Pain	Sham		N	83	1	45.1	50
		Tens		N	84			
		Acupuncture TENS		N	78			
		Tens	Biphasic	N	79			
Javadian et al. 2012 [115]	J Back Musculoskelet Rehabil	Exercise	Stabilization	N	30	3		
		Exercise	Various	N				
Jousset et al. 2004 [116]	Spine	Multidisciplinary	Various	N	43	4.75	41.4	30.2
		Exercise	Individualized	N	41		39.4	36.6
Kankaanpää et al. 1999 [55]	Spine	Exercise	Various	Y	30	12	39.8	36.7
		Control	Various	N	24		39.3	33.3
Kim et al. 2015 [117]	Clin Rehabil	Exercise	Contraction	N	27	2	29.7	100
		Control		N	26		28.6	
Kim et al. 2018 [118]	J Sport Rehabil	Exercise	Various	N	38	3	39.5	60.5
		Exercise	Stabilization	N	39		46.2	53.8
Kobayashi et al. 2019 [119]	Complement Ther Med	Shiatsu	Various	N	30	2	67.4	66.7
		Standard Care		N	29		68.3	62.1
Kogure et al. 2015 [120]	PLoS One	Spinal Manipulation	Various	N	90	6	60	60
		Sham	Various	N	89		59.6	64
Koldaş Doğan et al. 2008 [121]	Clin Rheumatol	Aerobics	Walking	N	19	1	37.1	78.9
		Physical Agents	Various	N	18		41.5	77.8
		Control		N	18		42.1	77.8
Laosee et al. 2020 [122]	Complement Ther Med	Massage	Pressure	N	70	3.45	68.2	77.1
		Massage	Pressure	N	70		69.1	71.4
Lawand et al. 2015 [123]	Joint Bone Spine	Exercise	Stretching	N	30	6	49.4	80.6
		Control		N	30		47.5	73.3
Lewis et al. 2005 [124]	Spine	Exercise	Various	Y	33	12	46.1	65
		Exercise	Individualized	Y	29		45.7	65
Ma et al. 2021 [125]	Ann Palliat Med	Physical Agents	Needling	N	30	12	47.7	50
		Massage	Swedish Massage	N	30		49.2	63.3
Macedo et al. 2014 [126]	Phys Ther	Exercise	Various	N	86	12	49.6	52.3
		Motor Control Exercise	Symptom Guided	N	86		48.7	66.3
Maggi et al. 2022 [127]	Aging Clin Exp Res	Kinesio Taping	No Tension	Y	57	3	66.8	71.93
		Control		Y	62		67.8	82.26
Mannion et al. 1999 [57]	Spine	Exercise	Various	Y	46	6	46.3	61
		Aerobics	Low-Impact	N	47		45.2	54
		Physical Agents	Various	N	44		43.7	55
Mannion et al. 2001 [56]	Spine	Exercise	Various	Y	44	12	46.3	61
		Aerobics	Low-Impact	N	43		45.2	54
		Physical Agents	Various	N	40		43.7	55
Marshall et al. 2008 [128]	Spine	Spinal Manipulation	High Velocity, Low Amplitude, Various	N	12	9	34.3	50
		Spinal Manipulation	High Velocity, Low Amplitude	Y	13		35.8	54
		Spinal Manipulation	Non Thrust, Various	N	12		33.9	50
		Spinal Manipulation	Non Thrust	Y	13		41.7	42
Martí-Salvador et al. 2018 [129]	Arch Phys Med Rehabil	Spinal Manipulation	Various	N	33	2	43.4	51.5
		Sham	Various	N	33		41.7	60.6
Masharawi et al. 2013 [130]	J Back Musculoskelet Rehabil	Exercise	Various	N	20	2	52.45	100
		Control			20		53.6	100
Matarán-Peñarrocha et al. 2020 [131]	Clin Rehabil	Exercise	Various	N	32	6	54.3	53.1
		Exercise		N	32		53.2	46.9
Meng et al. 2011 [132]	Clin J Pain	Back School		Y	181	12	50.2	65.2
		Back School		Y	163		49.5	63
Monticone et al. 2013 [133]	Clin J Pain	Exercise	Various	Y	45	12	49.0	60
		Control	Various	Y	45		49.7	55.6
Monticone et al. 2014 [58]	Eur Spine J	Motor Control Exercise	Stabilizing	Y	10	3	58.9	70
		Control	Various	N	10		56.6	40
Morone et al. 2011 [59]	Eur J Phys Rehabil Med	Back School	Various	Y	41	5	61.2	58.5
		Control		N	29		58.6	72.4
Murtezani et al. 2015 [60]	J Back Musculoskelet Rehabil	Mckenzie	Symptom Guided	Y	110	3	48.8	25.2
		Physical Agents	Various	N	109		47.5	61.5
Nambi et al. 2014 [134]	Int J Yoga	Yoga	Various	Y	30	6	44.3	63.3
		Control	Strenghtening, Stretching	Y	30		43.7	43.3
Natour et al. 2015 [135]	Clin Rehabil	Pilates	Various	N	30	2.96	47.8	80
		Control			30		48.1	76.7
O’Keeffe et al. 2020 [136]	J Sports Med	Cognitive Functional Therapy	Individualized	Y	106	10 to 10.5	47	77.4
		Exercise	Various	Y	100		50.6	70
Prommanon et al. 2015 [137]	J Phys Ther Sci	Back Care Pillow		N	26	3	38.5	42.3
		Control		N	26		39.7	50
Rittweger et al. 2002 [138]	Spine	Exercise	Extention	N	25	6	49.8	44
		Exercise	Vibration	N	25		54.1	52
Roche-Leboucher et al. 2011 [61]	Spine	Exercise	Various	y	68	12	40.8	32.4
		Exercise	Various	N	64		38.7	37.5
Ryan et al. 2010 [139]	Man Ther	Exercise	Various	Y	20	3	45.2	70
		Control		Y	18		45.5	61.1
Sahin et al. 2018 [140]	Turk J Phys Med Rehab	Physical Agents	Various	N	50	12	50.4	64
		Control	Stretching And Strenghtening	N	50		46.2	62
Saper et al. 2017 [62]	Ann Intern Med	Yoga	Various	N	127	9.2	46.4	56.7
		Aerobics	Various	N	129		46.4	69.8
		Education		Y	64		44.2	65.6
Segal-Snir et al. 2016 [141]	J Back Musculoskelet Rehabil	Exercise	Rotation	N	20	1	57.2	100
		Control		N	15		54.7	100
Sipaviciene et al. 2020 [142]	Clin Biomech	Exercise	Stabilization	N	35	3	38.3	100
		Exercise	Strengthening	N	35		38.5	100
Suh et al. 2019 [143]	Med	Stretching	Stretching	Y	13	1.5	53.5	61.5
		Walking Exercise	Walking	Y	13		54.2	84.6
		Spinal Stabilization	Stabilization	Y	10		57.4	60
		Spinal Stabilization	Stabilization, Walking	Y	12		54.8	66.7
Tavafian et al. 2011 [65]	Clin J Pain	Education		Y	97	6	44.6	73.2
		Control		N	100		45.9	83
Tavafian et al. 2014 [64]	Int J Rheum Dis	Education		Y	87	12	44.6	74.7
		Control		N	91		46.2	82.4
Uzunkulaoğlu et al. 2018 [144]	Turk J Phys Med Rehabil	Kinesio Taping With Tension	Traction	N	30	6	21.6	63.3
		Kinesio Taping Without Tension		N	30		21.3	63.3
Van Dillen et al. 2020 [145]	JAMA Neurol	Exercise	Various	N	74	12	42.4	68
		Exercise	Stretching, Strengthening	N	75		42.6	55
Verbrugghe et al. 2021 [146]	Int J Environ Res Public Health	Exercise	High-Intensity Strengthening	N	16	6	44.3	68.4
		Control	Moderate-Intensity Strengthening	N	13		44	68.4
Vollenbroek-Hutten et al. 2004 [147]	Clin Rehabil	Multidisciplinary	Various	Y	69	6	38.5	
		Control			73		39.5	
Werners et al. 1999 [148]	Spine	Tens		N	74	3	38.3	43
		Massage	Traction	N	73		39.2	49
Yang et al. 2021 [63]	J Bodyw Mov Ther	Pilates	Various	N	20	4.5	50.5	75
		Control		Y	19		47.9	79.0
Yeung et al. 2003 [149]	J Altern Complement Med	Exercise	Various	Y	26	3	55.6	80.8
		Exercise	Various	Y	26		50.4	84.6

*FU* follow-up, *LBP* lower back pain

### Study characteristics and results of individual studies

Data from 8152 patients were retrieved. The mean length of the follow-up was 6.2 ± 3.9 months. The mean length of symptom duration was 66.7 ± 51.6 months. The mean age of the patients was 46.7 ± 9.2 years. The generalities and demographics of the included studies are shown in Table 2.

**Table 2:** Baseline demographic

Endpoint	Non-Education (N = 5470)	Education (N = 3584)	P
Mean FU (*months*)	6.3 ± 5.7	8.9 ± 6.6	0.05
Mean age	45.6 ± 9.8	48.3 ± 7.3	0.05
Women (%)	61.4 ± 18.2	65.3 ± 15.0	0.1
Mean BMI	27.8 ± 8.5	26.2 ± 1.9	0.4
Symptoms (*months*)	66.5 ± 55.1	67.1 ± 46.6	0.9
VAS	5.4 ± 1.2	5.3 ± 1.3	0.7
RMQ	11.3 ± 6.9	10.1 ± 3.3	0.4

*FU* follow-up, *VAS* Visual Analogic Scale, *RMQ* Roland Morris Disability Questionnaire, *ODI* Oswestry Disability Index

### Baseline demographic

At baseline, the mean length of the follow-up and duration of symptoms, mean age and BMI, men:women ratio, VAS, and RMQ were comparable between groups (Table 2).

### Synthesis of results

At a mean of 7.4 ± 6.2 months, no difference was found in VAS (P = 0.8) and RMQ (P = 0.4) (Table 3).

**Table 3:** Main results

Endpoint	Non-education (N = 5470)	Education (N = 3584)	MD	SE	95% CI	*P*
VAS	3.5, 1.5	3.6, 1.3	0.1	0.2	− 0.36 to 0.56	0.8
RMQ	8.2, 8.8	6.6, 3.2	− 1.6	1.7	− 4.97 to 1.77	0.4

*VAS* Visual Analogic Scale, *RMQ* Roland Morris Disability Questionnaire, *MD* mean difference, *SE* standard error, *CI* confidence interval

### Meta-analysis

16 RCTs [50–65], including 2421 patients, compared education versus non-education in physiotherapy for chronic LBP and were included in the meta-analysis. Comparability was found at baseline between studies (Table 4).

**Table 4:** Baseline comparability of studies included in the meta-analysis

Endpoint	Non-education (N = 1261)	Education (N = 1160)	*P*
Mean age	44.7 ± 6.2	45.7 ± 5.9	0.6
Women:men	61.0 ± 14.5	60.9 ± 15.2	1.0
Mean BMI	26.7 ± 3.5	27.2 ± 3.5	0.8
Mean symptoms	71.4 ± 49.1	66.2 ± 43.4	0.8
Pain	5.4 ± 1.2	5.3 ± 1.0	1.0
RMQ	7.8 ± 1.9	7.3 ± 2.7	0.6
ODI	28.2 ± 6.5	32.6 ± 6.2	0.5

*VAS* Visual Analogic Scale, *RMQ* Roland Morris Disability Questionnaire, *ODI* Oswestry Disability Index

**Fig. 3:**
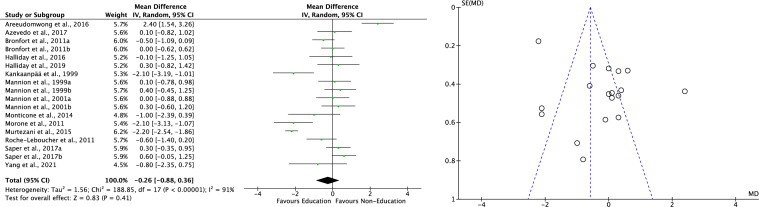
Meta-analysis of the comparison: pain scores

Fourteen RCTs [50–63] compared education versus non-education in pain scores with no difference (MD − 0.3; 95% CI − 0.88, 0.36; P = 0.4; Fig. 3, left). The Egger test found no statistically significant asymmetry of the funnel plots (P_Egger_ = 0.06), indicating acceptable data dispersion (Fig. 3, right).

**Fig. 4:**
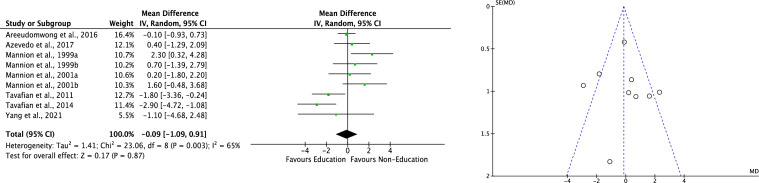
Meta-analysis of the comparison: RMQ

Seven RCTs [50, 51, 56, 57, 63–65] compared education versus non-education in RMQ with no difference (MD −0.09; 95% CI −1.09, 0.91; P = 0.9; Fig. 4, left). The Egger test found no statistically significant asymmetry of the funnel plots (P_Egger_ = 0.2), indicating acceptable data dispersion (Fig. 4, right).

## Discussion

The main findings of the present study show that education did not improve pain and disability in patients who undergo physiotherapy for chronic LBP. Our results are consistent with the existing literature, which observed no superiority of education in direct comparison to other interventions or only minor improvements in combination with other interventions [26, 66, 67].

The effects of different educational and physical exercise programs for LBP have been evaluated in multiple studies, with mixed results regarding improvements in QoL, pain level, disability and return to work [68, 69]. Previous reviews and meta-analyses have studied the influence of physiotherapy on LBP [4, 25, 29]. These studies have shown that physical activity improves pain, physical function, disability, and QoL with moderate certainty and no adverse events [4, 25, 29]. However, the overall evidence is low, primarily because of small sample cohort sizes, short follow-up periods and various definitions of physical activity [4]. A systematic review and meta-analysis [27] suggest that individual educational programs of 2.5 h in patients with LBP effectively increase return to work rates. Other interventions, including written material and shorter interventions, do not seem to reduce short- or long-term pain [27].

No available systematic review and meta-analysis investigated the combined effects of patient education and physiotherapy on the clinical outcome in patients with LBP. Pain is an important outcome measure in patients with LBP [70, 71]. The persistence of pain after interventions such as physiotherapy and education remains a challenge in the conservative management of LBP [72]. The present study suggests there is no advantage over education in combination with physiotherapy versus physiotherapy, only in reducing pain over 12 months. Our finding is supported by previous meta-analyses researching different non-interventional treatment options for LBP [25, 27, 73]. A systematic review and network meta-analysis investigated different treatments for acute and subacute mechanical non-specific LBP [25]; manual therapy was the most effective treatment, and education was the least effective. In a beforementioned meta-analysis [27], a 2.5 h educational intervention improves the return-to-work time but does not improve pain or disability. Low-quality evidence concluded that spinal manipulation plus advice slightly improved function at one week, without any difference in the long term [73].

RMQ is a short and straightforward method of self-assessment in patients with back pain [46, 74, 75], especially useful for following patients’ progress and combining different measures of function, such as disability and psychological factors [46, 74, 75]. An RMQ Score was provided in seven studies [57, 64, 65, 76–80]. There seem to be no advantages in education and physiotherapy regarding improving disability in the individual patient. As stated for the pain outcome measure, short-term disability does not seem to improve with educational interventions in addition to physiotherapy [25]. Physiotherapy improves physical function and disability with moderate certainty in chronic pain. At the same time, the included control groups included educational interventions [29]. This is supported by a recent meta-analysis [4] with moderate-certainty physiotherapy and exercises improved functional limitations compared to other conservative treatments. In the subgroup analysis, physiotherapy appeared more effective than advice or education alone [4].

The present study employed a stringent protocol for high-quality assessment of existing literature. To allow for comparability and avoid bias, strict inclusion criteria were used. Nonetheless, the results of this review should be interpreted against potential sources of bias, including the partially low numbers of participants ranging from 24 to 391 [81, 82]. Regarding a possible selection bias, all included studies used a randomised design for patient allocation. The low boundary of the follow-up period of 12 months limits a statement regarding long-term results. Multiple different definitions of physiotherapy were included, e.g. aerobic, strength, flexibility, range of motion, and core or balance training programmes, as well as yoga, Pilates, and tai chi, etc., and multiple forms of education ranging from oral interventions of different lengths to written material to psychotherapeutic interventions. Previous interventional and conservative treatments were rarely reported.

It is well known that education is more important in chronic patients who have developed psychosocial barriers to conservative treatment, such as fear of movement, low self-efficacy, and catastrophising [83, 84]. Educational interventions often aim to address cognitive-behavioural factors, such as fear-avoidance beliefs and catastrophising and the mechanisms through which these interventions influence pain and disability are not fully understood [85, 86]. However, given the lack of this information, patients who could have psychological prognostic conditions unfavourable to standard physiotherapy treatment were not analysed separately. This might limit the present study in identifying the real potential of education. Moreover, neurophysiological factors may also contribute to the persistence of pain and disability in LBP. A recent study demonstrated that various indices of central sensitisation can influence the ability of individuals with CLBP to self-manage their condition [87]. This should be addressed in future research and considered when analysing our results.

This suggests that addressing central sensitisation, a phenomenon in which the nervous system becomes hypersensitive to pain signals, may be crucial in improving the outcomes for some patients with CLBP.

Proper patient selection is pivotal for an educational program. However, there are no recommendations in this regard. Moreover, several studies have compared the efficacy of education in patients who have undergone different conservative therapies and physiographic approaches (e.g., active versus passive techniques). Finally, few studies reported data on ODI at the last follow-up; therefore, this endpoint was not considered in the analyses.

## Conclusion

The addition of education might not improve pain and disability in patients who undergo physiotherapy for chronic LBP. Further investigations should evaluate the potential of education in patients with chronic LBP who have developed psychosocial barriers to conservative treatment.

## Supplementary Information

Below is the link to the electronic supplementary material.

Supplementary file1 (DOCX 19 KB)

## Data Availability

The datasets generated during and/or analysed during the current study are available throughout the manuscript. No datasets were generated or analysed during the current study.
