# Supplementary material for: Impact of education in patients undergoing physiotherapy for lower back pain: a level I systematic review and meta-analysis
Source: Eur J Trauma Emerg Surg. 2025 Feb 19;51(1):113. doi: 10.1007/s00068-025-02788-9 (PMC11839871; doi:10.1007/s00068-025-02788-9)
Supplement: Supplementary file 1 — Supplementary file1 (DOCX 19 KB) [file 68_2025_2788_MOESM1_ESM.docx]

**Research Question:**

Impact of education in patients undergoing physiotherapy for lower back pain: a level I systematic review and meta-analysis

**Concept 1: Disease**

**Keywords:**

low back pain

LBP

non-specific low back pain

mechanical low back pain

chronic low back pain

**Mesh:**

"Low Back Pain"[Mesh]

"Low Back Pain/therapy"[Mesh]

"Low Back Pain/rehabilitation"[Mesh]

**Concept 2: Therapy**

**Keywords:**

physiotherapy

physical therapy

manual therapy

Massage

Mobilization

spinal manipulation

Lumbar stabilization

Active and passive stretching

exercises

muscle exercises

motor control exercises

strengthening exercises

stabilizing exercises

functional resistance training

muscle strength training

Back school

McKenzie

Acupuncture

Yoga

**Mesh:**

"Physical Therapy Modalities"[Mesh]

"Transcutaneous Electric Nerve Stimulation"[Mesh]

"Musculoskeletal Manipulations"[Mesh]

"Massage"[Mesh]

"Manipulation, Spinal"[Mesh]

"Muscle Stretching Exercises"[Mesh]

"Exercise"[Mesh]

"Exercise Therapy"[Mesh]

"Acupuncture"[Mesh]

"Acupuncture Therapy"[Mesh]

"Yoga"[Mesh]

**Concept 3: Others**

**Keywords:**

Education approaches

Non-education approaches

**Mesh:**

"Patient Education as Topic"[Mesh]

**Searching Strategy**

"Low Back Pain"[Mesh] OR "Low Back Pain/therapy"[Mesh] OR "Low Back Pain/rehabilitation"[Mesh] OR "Spine"[Mesh] OR Low back pain OR LBP OR non-specific Low back pain OR mechanical Low back pain OR chronic Low back pain OR spine

**AND**

"Physical Therapy Modalities"[Mesh] OR physiotherapy

**AND**

"Musculoskeletal Manipulations"[Mesh] OR "Massage"[Mesh] OR "Manipulation, Spinal"[Mesh] OR "Muscle Stretching Exercises"[Mesh] OR "Exercise"[Mesh] OR "Exercise Therapy"[Mesh] OR "Acupuncture"[Mesh] OR "Acupuncture Therapy"[Mesh] OR "Yoga"[Mesh] OR physical therapy OR manual therapy OR massage OR mobilization OR spinal manipulation OR lumbar stabilization OR active and passive stretching OR exercises OR muscle exercises OR motor control exercises OR strengthening exercises OR stabilizing exercises OR functional resistance training OR muscle strength training OR Back school OR McKenzie OR Acupuncture OR Pilates OR Yoga

**AND**

"Patient Education as Topic"[Mesh] OR education approaches OR non-education approaches

**SUMMARY**

((("Low Back Pain"[Mesh] OR "Low Back Pain/therapy"[Mesh] OR "Low Back Pain/rehabilitation"[Mesh] OR "Spine"[Mesh] OR Low back pain OR LBP OR non-specific Low back pain OR mechanical Low back pain OR chronic Low back pain OR spine) AND ("Physical Therapy Modalities"[Mesh] OR physiotherapy)) AND ("Musculoskeletal Manipulations"[Mesh] OR "Massage"[Mesh] OR "Manipulation, Spinal"[Mesh] OR "Muscle Stretching Exercises"[Mesh] OR "Exercise"[Mesh] OR "Exercise Therapy"[Mesh] OR "Acupuncture"[Mesh] OR "Acupuncture Therapy"[Mesh] OR "Yoga"[Mesh] OR physical therapy OR manual therapy OR massage OR mobilization OR spinal manipulation OR lumbar stabilization OR active and passive stretching OR exercises OR muscle exercises OR motor control exercises OR strengthening exercises OR stabilizing exercises OR functional resistance training OR muscle strength training OR Back school OR McKenzie OR Acupuncture OR Pilates OR Yoga)) AND ("Patient Education as Topic"[Mesh] OR education approaches OR non-education approaches)
